# Mechanical deformation inhibits growth and migration of *S. aureus* within submicrometer channels

**DOI:** 10.1128/mbio.00194-26

**Published:** 2026-04-23

**Authors:** Kelsey G. DeFrates, Junsung Lee, Gissell Jimenez, Jae Won Hwang, Mariana G. Pinho, Christopher J. Hernandez

**Affiliations:** 1 Department of Orthopaedic Surgery, University of California 540623, San Francisco, California, USA; 2 Sibley School of Mechanical and Aerospace Engineering, Cornell University 423500, Ithaca, New York, USA; 3 Instituto de Tecnologia Química e Biológica António Xavier, Universidade NOVA de Lisboa, Oeiras, Portugal; 4 Department of Bioengineering and Therapeutic Sciences, University of California 115280, San Francisco, California, USA; 5 Department of Bioengineering, University of California 224036, Berkeley, California, USA; 6 Biohub, San Francisco, California, USA; Universite de Geneve, Geneva, Switzerland

**Keywords:** *Staphylococcus aureus*, microfluidics, osteomyelitis

## Abstract

Bacteria colonize surfaces in the environment and can also penetrate tissues and materials by entering micro- and nano-scale cracks and pores. *Staphylococcus aureus* has been observed within nanoscale channels in bone that are 2–3 times smaller than cell diameter. Inside the bone, bacteria are protected from host immunity and systemic antibiotics, potentially contributing to chronic and recurrent infections. The physical mechanisms that enable bacteria to enter channels smaller than the cell width are unclear. It has been proposed that bacteria traverse narrow passages through division, such that daughter cells form within small channels and proliferate in chains down the channel length. Here, we use microfluidics to test the idea that *S. aureus* can traverse submicrometer channels through growth. We examined the net migration of growing cell chains within tapered nanochannels (width ~1.5–0.3 μm). We found that proliferation can facilitate migration, but only to cell deformations of 600 nm (65% cell width). Below 600 nm, mechanical confinement significantly slows or completely inhibits division in single cells. Interestingly, growth arrest occurs independent of the Z-ring assembly and is unrelated to the initial orientation of the division plane. Thus, our findings suggest that it is unlikely for *S. aureus* to traverse nanoscale channels via division.

**IMPORTANCE:**

Bacteria that colonize materials and tissues within the body can be difficult to remove, even with thorough cleaning and application of antibiotics. Recent studies show that bacteria not only colonize the surfaces of tissues in the body but can also squeeze into naturally occurring pores and channels and thereby gain protection from immune cells and antibiotics. Here, we ask how physical forces and cell growth might enable bacteria to enter small pores within materials. We use microfluidic devices to study the growth and migration of the human pathogenic bacteria, *S. aureus*.

## OBSERVATION

Bacterial colonization of materials and biological tissues is a significant challenge in human health. Many studies have focused on understanding and preventing the growth of organisms on the surfaces of materials and devices. However, it is also possible for bacteria to enter the materials by traversing narrow channels or pores in biological tissues or synthetic materials. For example, several recent studies have shown that, when infecting bone, *Staphylococcus aureus* is capable of colonizing naturally occurring channels within the bone matrix called canaliculi. Canaliculi range from 300 to 900 nm in width, requiring *S. aureus* to deform to one-third of its original cell size (1–5). The ability of *S. aureus* to colonize canaliculi is thought to be a major contributor to treatment failure and chronic infection, as bacteria become protected from immune cell clearance and antibiotic therapy within nanochannels (1–3). Despite clinical relevance, how *S. aureus* or other bacteria may penetrate submicrolmeter channels remains underexplored.

**Fig 1:**
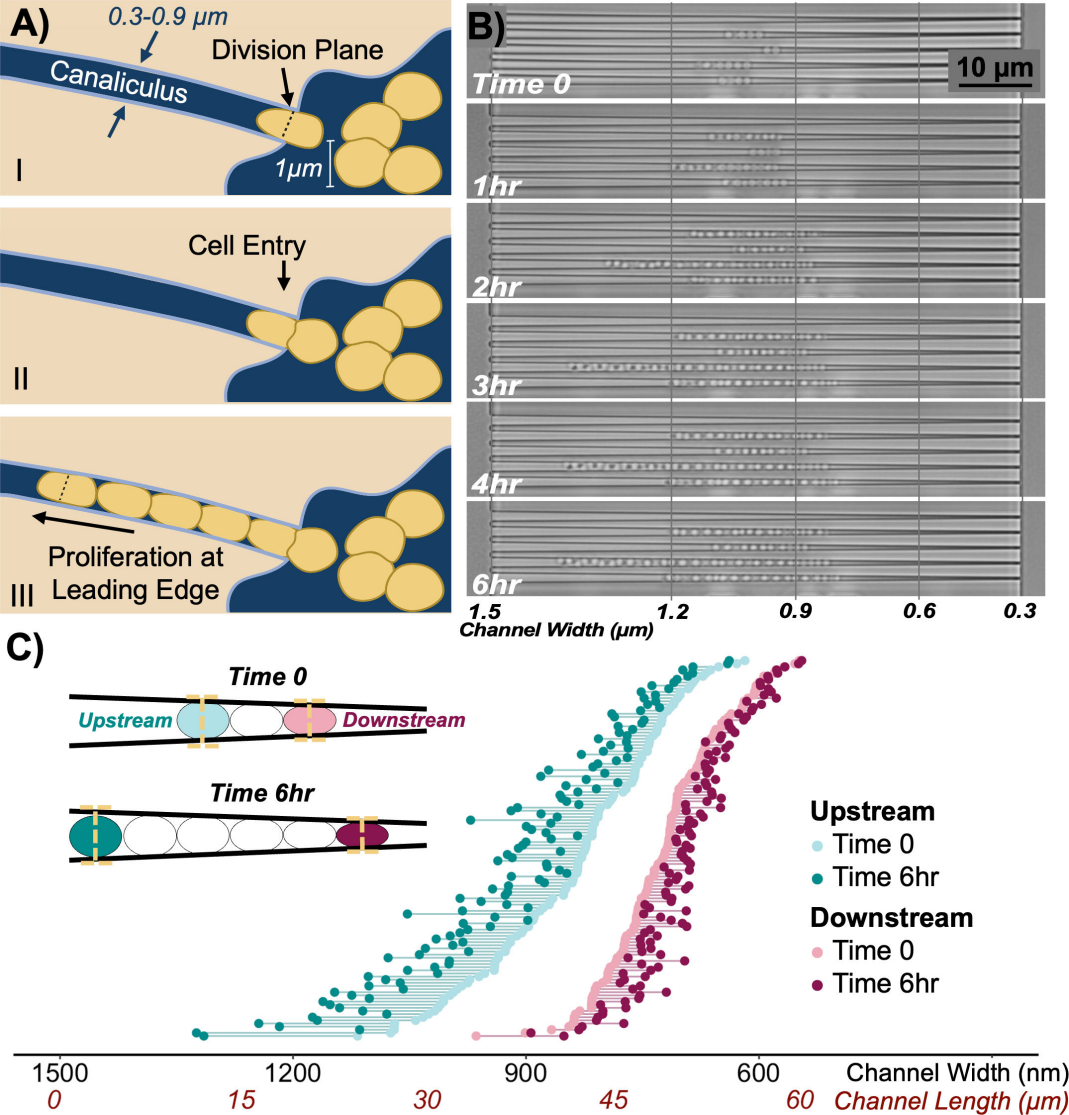
Illustration of the proposed mechanism of entry of *S. aureus* into bone through growth (**A**). The septal plane forms perpendicular to the channel opening (I), propelling one daughter cell into the canaliculus (II). Inside the channel, cells continue to divide, leading to migration down the channel length (III). Representative image of cell migration due to the growth of cell chains loaded concurrently at time 0, illustrating initial growth in both directions. At later time points, however, net migration toward the wider side of the taper is greater than migration toward the narrow end (**B**). Net migration of the upstream and downstream ends of cell chains after 6 h is shown in terms of change in distance along the channel width and length (**C**). Each point represents the upstream (teal) and downstream (pink) position of a single chain (*n* = 121 chains).

Microfabricated systems and devices have been used to elucidate mechanisms of bacteria transport and migration in different environments (6–9). These studies have shown that flagellar motility is limited, as channel dimensions approach cell width (6, 9). However, both motile and non-motile bacteria have the capacity to traverse channels smaller than cell width through growth and division. Migration through division is achieved when a single bacterium becomes lodged in a channel opening and divides, eventually giving rise to a chain of growing cells that extends the length of the channel (6). Whole cell stiffness likely influences the ability of bacteria to migrate via cell division; *Escherichia coli* (undeformed width 0.8 µm) can traverse channels as small as 0.4 µm in width via growth; however, the stiffer *Bacillus subtilis* (undeformed width 0.9 µm) cannot divide through passages smaller than 0.75 µm (6, 10). It has been proposed that *S. aureus* can also enter canaliculi in the bone through cell division (1). To facilitate migration, alignment of the septal plane across the channel width may anchor growing cells, leading to daughter cells being propelled forward into the nanochannel (Fig. 1A) (1). Evidence suggests that *S. aureus* may also preferentially sense and divide into canaliculi through a process known as durotaxis (1, 5, 11). In support of this hypothesis, *S. aureus* was found to propagate through 0.5 µm pores within a 0.4 µm thick silicon membrane (1, 5, 11). However, a thin, porous membrane does not recapitulate the channel-like geometry of canaliculi or allow for repeated cell divisions, which would be needed to traverse canaliculi. Therefore, in this study, we used microfluidics to examine the effects of mechanical deformation on *S. aureus* growth and migration within nanochannels.

We used a microfluidic device initially developed by our laboratory to study the biomechanics and mechanobiology of bacteria (10, 12–14). The device, which we call the “extrusion loading device,” is manufactured from silica glass using Deep UV lithography and consists of a series of tapered channels that narrow in width from ~1.5 µm to 0.3 µm (Fig. S1). When a suspension of bacteria is flowed into the device, individual bacteria are trapped within the tapered channels by a pressure differential (ΔP) generated between the channel inlet and outlet (12). Greater pressure differences cause cells to travel further into the channel and experience greater deformation. To subject bacteria to a range of deformations, four distinct loading pressures are generated within one device by connecting sets of closely spaced channels in parallel. Prior work has verified that inside channels, bacteria receive sufficient nutrients to support growth and viability for several hours (12).

Previously, we used the extrusion loading device to investigate the transport and deformation of *S. aureus* within submicrometer channels as a function of applied fluid pressure (13). Here, we apply a similar range of pressures (1–6.5 kPa) to deform the methicillin-resistant *S. aureus* COL strain to canaliculi-like widths. We note that our microfluidic device may not fully replicate the *in vivo* bone microenvironment, which features tortuous cylindrical channels lined with extracellular matrix proteins that may influence *S. aureus* adhesion and migration. Additionally, planktonic cells are used to initially populate the device, which may differ from biofilm-associated cells often found at the surface of orthopedic implants and tissue *in vivo*. Extracellular polymeric substances secreted by biofilm-associated cells may influence their migration and transport within canaliculi, for example, by influencing friction between the canaliculus and cell wall, which is not recapitulated in our experiment. Nonetheless, the device and current experimental conditions allow us to study the effects of mechanical deformation on *S. aureus* proliferation and migration, which has not been previously reported. To better recapitulate cell growth within canaliculi, we first analyzed the net migration of growing chains of cells (3+), trapped within the device at time 0. We observed that over time, chains extended both upstream (toward the wider end of taper) and downstream (toward the narrower end of the taper) (Fig. 1B). However, after 6 h, the total net migration of the upstream chain end, where cells were less deformed, was greater than that of downstream cells (Fig. 1C). Movement of downstream chain ends also slowed once channel width approached 600 nm, suggesting that significant mechanical deformation may inhibit cell division and prevent migration via growth.

**Fig 2:**
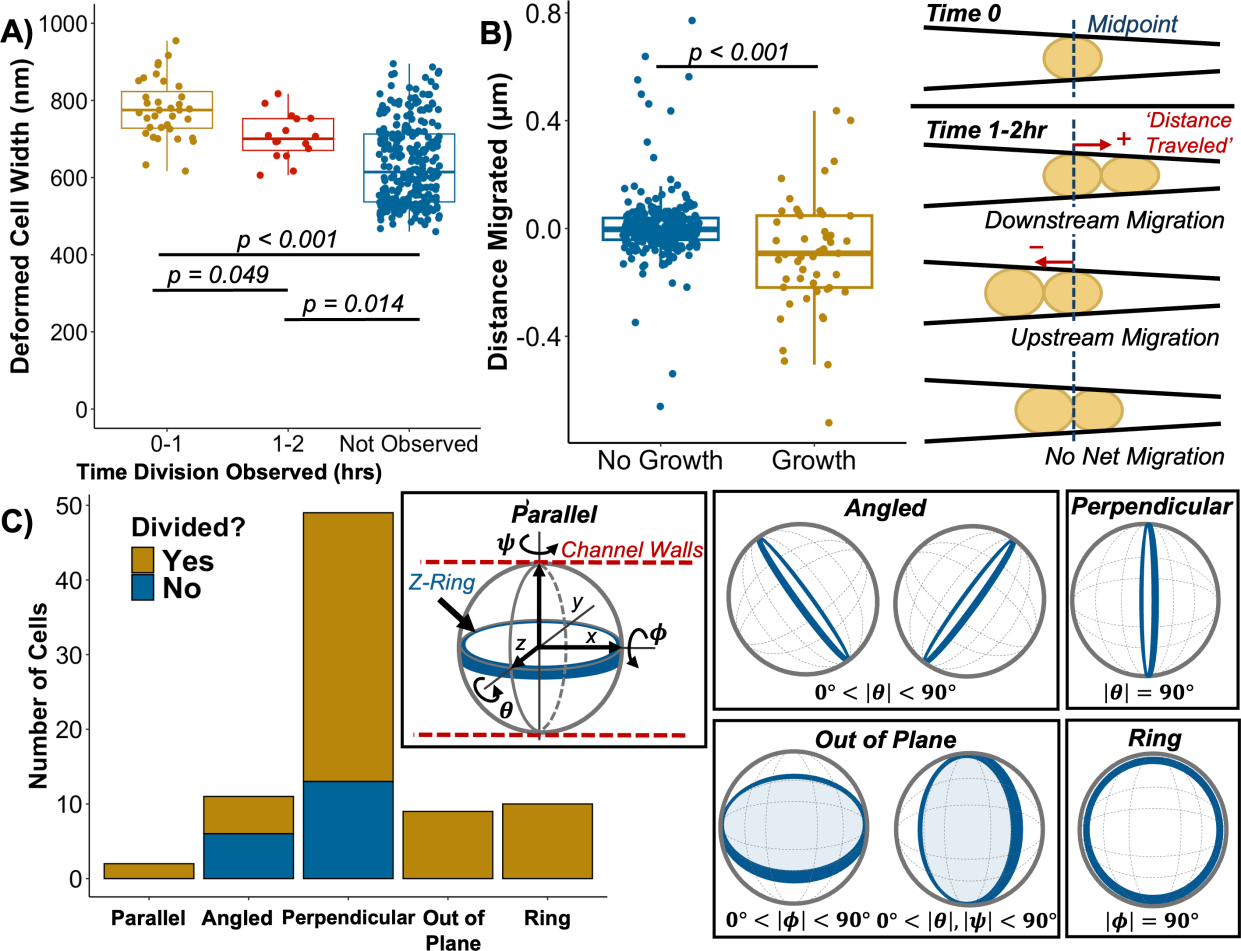
Deformed cell width is reported for single cells trapped within nanochannels, which did or did not divide over the course of 3 h (**A**). Generally, cells that were more deformed took longer to divide. Average deformed width of cells that divided during the first interval hour within the microfluidic device was 778 ± 77 nm (mean ± sd, *n* = 35) versus 706 ± 59 nm (*n* = 16). Cells that did not divide were the most deformed, with an average width of 634 ± 104 nm (*n* = 271). The distance migrated for cells that divided within the extrusion loading device (*n* = 51) and for cells that did not divide (*n* = 271) is shown with a schematic illustration of the analysis method (**B**). To determine if the initial orientation of the Z-ring contributed to delayed growth in deformed cells, the placement of the division septum relative to the channel walls was categorized in COL EzrA-sGFP cells (*n* = 80) (**C**). A schematic illustrating possible Z-ring orientations for cells within the device is shown. The “parallel” orientation refers to alignment of the Z-ring across the *x*-axis of the cell, and “perpendicular” orientation across the *y*-axis. If the parallel orientation were to be turned approximately +/−90° about the *x*-axis (ϕ), the Z-ring would then become perpendicular to the field of view and appear as a ring around the boundary of the cell. If turned less than 90° in either direction about the *x*-axis, the Z-ring becomes “out of plane,” leading to non-uniform fluorescence across the cell body. Statistics are based on a one-way ANOVA with Tukey HSD for panel **A** and a Student’s *t*-test for panel **B**.

To better understand how mechanical confinement may slow or inhibit growth, we examined the divisions of single cells trapped in channels. Across three biological replicates, we analyzed 322 individual cells initially trapped at widths ranging from 955 to 460 nm within channels (undeformed cell width: 931 ± 41 nm, mean ± SD) (Fig. S2). We observed that deformation slowed *S. aureus* division; cells that were more deformed were less likely to divide in the first hour (Fig. 2A). Cells that did not divide at all over the course of the experiment experienced the greatest deformation (width 634 ± 104 nm); no divisions were detected in cells measuring less than 606 nm in width. If *S. aureus* preferentially senses and divides into canaliculi as proposed (4, 8, 15), we hypothesized that division would result in the net migration of cells toward the narrower end of the channel. However, for single cells that divided within the channel (*n* = 51), we found that growth and division had a marginal effect on cell positioning. On average, dividing cells migrated only 97 ± 215 nm and showed a slight bias (56% of cells studied) to migrate upstream toward the wider end of the channel (Fig. 2B). This suggests that *S. aureus* in bone is unlikely to preferentially divide into canaliculi and that deformation below ~65% cell width severely inhibits *S. aureus* growth.

We hypothesized that growth arrest within the extrusion loading device may be related to the initial orientation and/or later formation of the division septum. Thus, we visualized assembly and localization of the Z-ring for cells within the extrusion loading device using a COL strain expressing a functional fusion of the FtsZ interaction partner, EzrA, to superfast green fluorescent protein (sGFP) (*n* = 164 total cells from three replicate experiments) (15). Again, we observed that cells subjected to higher degrees of mechanical deformation took longer to divide or did not divide at all, even after 8 h of observation (Fig. S3). We suspected that division may be physically blocked if cells were to become trapped within the device in an orientation that aligned the Z-ring parallel to the channel walls (Fig. 2D; Fig. S4A). When we quantified Z-ring orientation in all cells with visible septa at time 0 (*n* = 80), however, we found few cells (1.2%) with Z-rings in the parallel orientation (Fig. 2C). Instead, the majority of Z-rings (61%) were found to be aligned perpendicular to the channel walls, which is the orientation least likely to impair division. This preferred orientation is likely due to the fact that *S. aureus* elongates slightly during formation of the division septum (16). Cells will then align longitudinally when entering channels, so that the shorter axis, overlapping the septum, becomes perpendicular to the walls. We also observed instances where the Z-ring appeared angled relative to the channel walls, as well as oriented perpendicular or out of plane to the field of view (Fig. 2C). Division was observed for all Z-ring orientations. Thus, stalled or inhibited cell growth in the extrusion device appears to be unrelated to the initial orientation of the division plane.

Next, we sought to determine if mechanical confinement affected division by preventing the formation of new Z-rings after initial cell loading. We identified cells within the extrusion loading device that did not exhibit a detectable Z-ring at time 0 (*n* = 84) and instead showed uniform low-intensity fluorescence across the entire cell body (Fig. S4A). We note that cells that were deformed more than 30% of their original width were more likely to be classified as having undetectable Z-rings. Thus, it is also possible that large deformations prevent accurate visualization of the Z-ring due to image resolution (Fig. S4B). However, within a deformation range where Z-rings were reliably detected in other cells (<30% deformation), we found a significant number of cells with undetectable Z-rings at time 0 (*n* = 49), suggesting that Z-ring assembly had not yet commenced. Notably, 41% of cells with undetectable Z-rings at time 0 still divided within 8 h, suggesting that mechanical confinement did not impair later formation of the division septum. Time-lapse imaging verified that Z-rings did form in cells at later time points (Fig. S5). Interestingly, we observed instances where Z-rings formed in greatly deformed cells that did not divide during the experiment. These non-dividing cells also appeared to elongate within the device, suggesting that viability was maintained. Thus, the mechanism by which mechanical deformation slows or impairs *S. aureus* division remains unresolved but appears to be unrelated to septum assembly or cell viability. Bacteria exhibiting delayed growth have previously been shown to exhibit higher tolerance to antibiotics and are implicated in the emergence of dormant, “persister” cell populations (17). Therefore, if mechanical deformation within canaliculi causes *S. aureus* to adopt a similar slow-growing state *in vivo*, this may further contribute to infection recalcitrance and treatment failure.

Given our observation of impaired growth in deformed cells, coupled with our findings that proliferation does not lead to significant migration of single cells or cell chains in channels measuring less than 600 nm in width, we conclude that *S. aureus* is unlikely to enter canaliculi in bone through growth and division characteristic of durotaxis. As *S. aureus* is non-motile, external forces would be required to transport cells into canaliculi. Therefore, we speculate that *S. aureus* transport in bone may be facilitated by fluid pressures *in vivo*. Fluid pressures generated in bone during normal physical activity have been reported to be as high as 8–20 kPa (18). Based on prior studies investigating *S. aureus* deformation and transport as a function of applied pressure, we estimate that only 9 kPa of pressure would be needed to force cells into channels measuring 300 nm, the smallest width of canaliculi (13). Thus, the ability of bacteria to colonize canaliculi may be more closely related to physical loads on the bone and the mechanical properties of the bacteria rather than growth. Our work also provides insight into how bacteria may colonize micro- and nano-scale cavities in other biological and synthetic materials where cells can become protected from predation, sterilization, and antimicrobial therapies (19, 20).

### Cell culturing

*S. aureus* COL strains were grown overnight (18 h) in tryptic soy broth (TSB) (Millipore Sigma) at 37°C with 200 rpm shaking. The overnight culture was diluted 1:200 in TSB and grown for 2–3 h at 37°C until OD_600_ ~0.3 to reach the exponential phase. Construction of the COL strain expressing EzrA-sGFP used in this study can be found in reference 15.

### Microfluidic device manufacturing

Microfluidic device manufacturing was done using Deep UV photolithography as previously described (10, 12–14). Briefly, fused silica wafers (100 mm diameter and 500 µm thick, WF3937X02031190, Mark Optics, Santa Ana, CA, USA) were coated with ~55 nm of chrome using the AJA Sputter Deposition Tool (AJA International, Scituate, MA, USA). An ~60 nm coat of anti-reflective coating (ARC, DUV 42P, Brewer Science, Rolla, MO, USA) and ~510 nm coat of photoresist (UV210, MicroChem, Westborough, MA, USA) were then applied using the Gamma Automatic Coat-Develop Tool (Suss MicroTec Gamma Cluster Tool, Garching, Germany). The custom microfluidic device pattern was transferred to the wafer using the ASML Deep UV stepper (Veldhoven, the Netherlands). The photoresist was then developed using the Gamma Automatic Coat-Develop Tool, and the pattern was transferred from the photoresist to the anti-reflective coating using plasma etching in the Oxford 82 Tool (Oxford, Abingdon, UK). The pattern was transferred to the chrome layer using the Plasma-Therm 770 ICP tool (Plasma-Therm St. Petersburg, FL, USA). Oxygen plasma cleaning by the Oxford 82 Tool was performed to remove residual coating before the pattern was finally transferred to the silica wafer using the Oxford 100 Tool (Oxford, Abingdon, UK). Any remaining chrome was removed using a wet chemical bath. Through-holes were laser-etched at the microfluidic device inlets and outlets using a Versalaser (VLS3.50, Universal Laser Systems, Scottsdale, AZ, USA) to establish inlet and outlet holes. All fabrication steps were performed within the cleanroom of the Cornell NanoScale Facility Science and Technology Facility (Ithaca, NY, USA).

To verify that the pattern was successfully transferred, device feature dimensions were characterized using atomic force microscopy (Veeco Icon Bruker, Billerica, MA, USA), profilometry (P-7, KLA Inc, Milpitas, CA, USA), and scanning electron microscopy (Zeiss Ultra 55 SEM microscope, Oberkocken Germany). Devices used in this study had an average depth ranging from 1.0 to 1.2 µm, a taper inlet width of 1.73–1.59 µm, and an outlet width of 0.46–0.315 µm.

Successfully patterned wafers were bonded to silica cover wafers (100 mm diameter and 170 µm thick) (WF3937X0073119B Mark Optics, Santa Ana, CA, USA) after MOS/RCA cleaning by hand bonding and nitrogen annealing (5 h, 1,100°C).

### *S. aureus* loading within the microfluidic device

To load *S. aureus* cells into the microfluidic device, fluid pressure was supplied by a PneuWave Pump (CorSolutions, Ithaca, NY, USA). The pump was attached to the microfluidic device inlet using PEEK tubing (Idex 360 µm OD × 150 µm ID, Lake Forest IL, USA) that was fed through a magnetic connector lever arm (Fluidic Indexing Probe, CorSolutions, Ithaca NY, USA). A rubber gasket (N-123-03 IDEX, Lake Forest, IL, USA) was also included at the end of the tubing to prevent leakage. Before loading cells, the tubing was sterilized by running 10% bleach, followed by 70% ethanol through the pump for 15 min. TSB was then flowed through the tubing to waste for 20 min before attaching to the device. The device was wet with TSB for 30 min. The tubing was then disconnected from the device, and the cell suspension was flushed through for 15 min into waste. The tubing was then reattached to the device, and 60 or 80 kPa of pressure was applied to the load cells within tapered channels. Two different loading pressures were used to ensure that the observed results were the effect of cell deformation by the channel walls, rather than flow rate or hydrostatic loading. We observed no differences in growth kinetics at the two loading pressures, in line with published works from our group showing that changes in bacterial physiology and function within the extrusion loading device are more strongly correlated to differential fluid pressures across individual tapers and/or the magnitude of mechanical deformation experienced by cells within the device (12, 14). Media flowed continuously through the device for the remainder of the experiment. To observe cell loading and division, the device was mounted on an Olympus IX83 inverted microscope (Evident Scientific, Waltham, MA, USA) equipped with a motorized stage (Märzhäuser Wetzlar SCAN IM, Wetzlar, Germany) and Okolab Cage Incubator set to 37°C (H201-Enclosure with temperature control unit, Okolab, Sewickley PA, USA). Immediately after loading and hourly for 3–8 h, brightfield images were taken of cells in individual tapers using a 100× objective (UPLXAPO100X0, Evident Scientific, Waltham, MA, USA).

For experiments investigating Z-ring orientation and/or formation, a similar procedure was followed with COL EzrA-sGFP cells suspended in TSB or a 50:50 mixture of TSB:PBS to reduce background fluorescence for imaging. We observed no effect of media composition on cell division trends relative to deformation (Fig. S3). Brightfield and fluorescence images at 475 nm excitation were collected every 15 min for the first 2 h after cell loading and then hourly for up to 8 h, using the Olympus IX83 previously described, equipped with a X-Cite Novem Channel LED Illumination System (Excelitas Technologies Corp, Pleasanton, CA, USA).

### Image analysis

A custom MATLAB (v. 2023b, Mathworks, Natick, MA, USA) script was developed to measure cell width and distance traveled within the microfluidic device (10, 12–14). The script requires user input to identify regions of interest within brightfield images containing cells. Horizontal and vertical line profiles are then taken within the region. The distance between the midpoints in curves from the horizontal and vertical profiles is used to estimate cell length and width, respectively (Fig. S6). Users then identify the channel end, and the distance between the midpoint of the cell and the outlet is calculated. The distance cells traveled in the channel was also used to calculate the expected deformed width based on the channel geometry. To determine the distance migrated for cell chains, the starting position of cells at the upstream and downstream edge of the chain was taken at time 0 and compared to time 6 h. For single-cell analysis, the midpoint of dividing cells was taken at time 0 or time 1 h if cells divided during the first or second hour of the experiment, respectively. After cell division, the midpoint of the daughter cell pair was then determined and compared to the midpoint of the original cell. If cells did not divide over the course of the experiment, the change in distance for the single cell between time 0 and time 1 h was determined. For all analyses, a frame of reference was established so that when cells moved downstream (toward the narrower end of the taper), this resulted in a net positive change in distance, while movement upstream (toward the wider end) resulted in a negative change.

To determine the orientation of the Z-ring in COL EzrA-sGFP cells, once cell boundaries were identified in MATLAB, background intensity was used to threshold fluorescent images in this region to isolate the Z-ring. The “regionprops” function within the MATLAB Image Processing Toolbox was applied to measure the angle between the *x*-axis and the major axis for the threshold object. Angles ranged from −90° to 90°, and absolute values (*a*) were used to classify Z-ring orientations as follows: parallel = 0 < *a* ≤ 22.5°, angled = 22.5° < *a* ≤ 67.5°, and perpendicular = 67.5° < *a*. Z-rings oriented perpendicular to the plane of view were manually identified when a fluorescent ring was observed around the boundary of the cell. Z-rings were categorized as “out of plane” by users if fluorescence appeared non-uniform across the cell body. A Z-ring was determined to be “undetectable” when uniform and/or little to no fluorescence was observed across the entire cell.

To determine the undeformed cell width, cells residing near the inlet of the microfluidic device that were not transported into tapered channels were imaged, and the average width was calculated using the MicrobeJ plugin on ImageJ.

### Microfluidic device hydraulic circuit pressure calculations

To determine the fluid pressure at the tapered channels within the microfluidic devices, we performed hydraulic circuit calculations using a custom script in MATLAB (v. 2023b, Mathworks, Natick, MA, USA) as described (10, 14). In the analysis, the Hagen-Poiseuille law (equation 1) is used to determine the pressure drop, Δ*P*, across each channel with flow rate *Q* and hydraulic resistance, *R_h_*.


(1) $$∆P=QR_{h}$$

To calculate *R_h_*, Poiseuille flow (equations 2 and 3) was used when the ratio of the channel width to height was small (<20), while Plane Poiseuille flow (equation 4) was used when width/height was larger (>20). In each case, hydraulic resistance is a function of the fluid viscosity, µ (assumed water = 8.9e−4 Pa s), the length of the channel, *L*, the cross-sectional area of the channel, *A*, and the hydraulic radius, *r*. For a rectangular channel, *r* is calculated from the perimeter of the channel cross-section, *P*, and channel height, *H*.


(2) $$R_{h}=\frac{8\mu L}{Ar^{2}}$$

(3) $$r=\frac{2A}{P}$$

(4) $$R_{h}=\frac{12\mu L}{AH^{2}}$$

To determine *R*_Total_, the total hydraulic resistance across the device *R_h_* values for all channels were combined. When channels were connected in parallel, *R*_Total_ was calculated using equation 5 for *n* number of channels.


(5) $$R_{Total}=\frac{1}{R_{1}}+\frac{1}{R_{2}}...+\frac{1}{R_{n}}$$

Channels connected in series were combined using equation 6.


(6) $$R_{Total}=\;R_{1}+\;R_{2}\ldots +R_{n}$$

### Statistical analysis

One-way ANOVA with *post-hoc* Tukey HSD or Student’s *t*-test was used to compare groups when appropriate. Statistical analyses were performed with a significance level of α = 0.05. Data were analyzed using RStudio.

## SUPPLEMENTAL MATERIAL

Supplemental Figures mbio.00194-26-s0001.pdf Fig. S1 to S6.

ASM does not own the copyrights to Supplemental Material that may be linked to, or accessed through, an article. The authors have granted ASM a non-exclusive, world-wide license to publish the Supplemental Material files. Please contact the corresponding author directly for reuse.
