# Supplementary material for: Mechanical deformation inhibits growth and migration of S. aureus within submicrometer channels
Source: mBio. 2026 Apr 23;17(5):e00194-26. doi: 10.1128/mbio.00194-26 (PMC13170230; doi:10.1128/mbio.00194-26)
Supplement: Supplemental Figures — Fig. S1 to S6. [file mbio.00194-26-s0001.pdf]

*Supplemental Information for:*

# Mechanical deformation inhibits growth and migration of *S. aureus* within submicron channels

Kelsey G. DeFrates[a], Junsung Lee[a,b*], Gissell Jimenez[a], Jae Won Hwang[a], Mariana G. Pinho[c], Christopher J. Hernandez[a,b,d,e,f#]

[a]Department of Orthopaedic Surgery, University of California, San Francisco, California, United States

[b]Sibley School of Mechanical and Aerospace Engineering, Cornell University, Ithaca, New York, United States

[c]Instituto de Tecnologia Química e Biológica António Xavier, Universidade NOVA de Lisboa, Oeiras, Portugal

[d]Department of Bioengineering and Therapeutic Sciences, University of California, San Francisco, California, United States

[e]Department of Bioengineering, University of California, Berkeley, California, United States

[f]Biohub, San Francisco, California, United States

Running Head: How bacteria traverse nanoscale channels

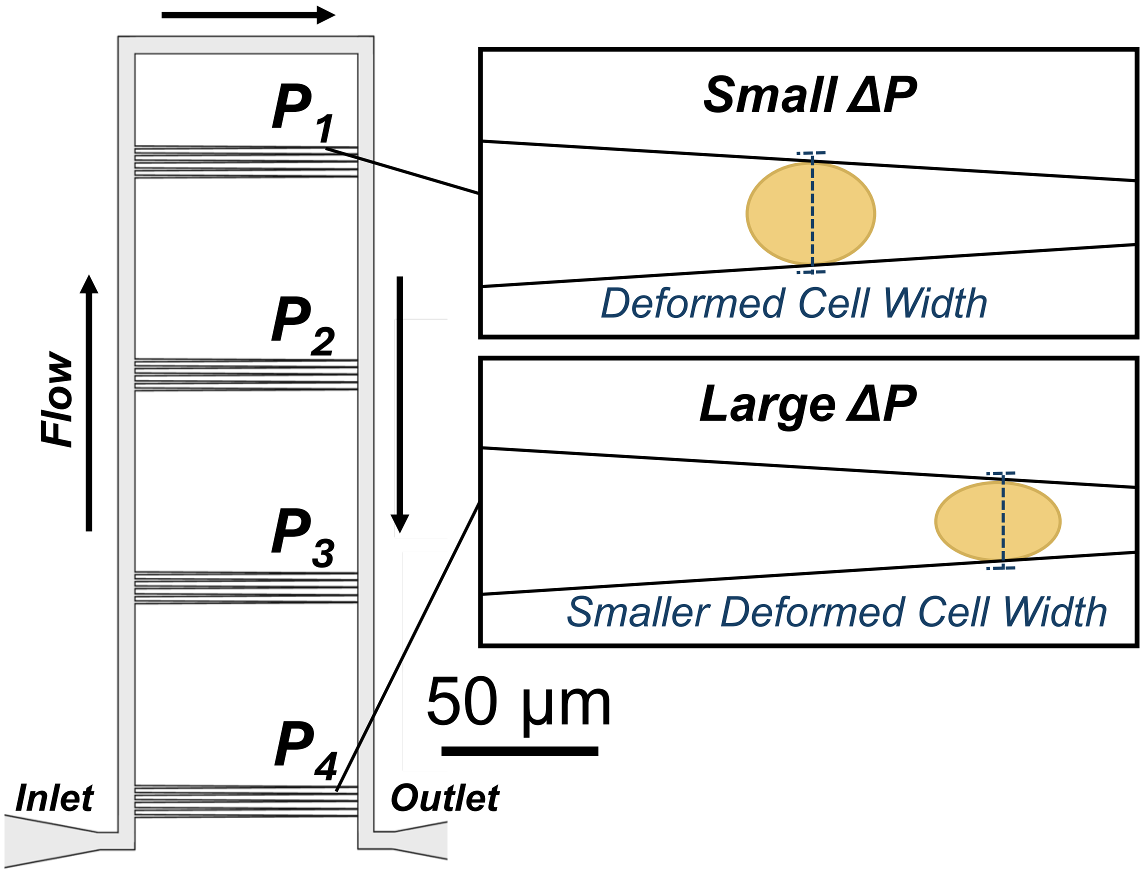


**Figure S1.** The extrusion loading device is shown with tapered channels connected in parallel to generate 4 distinct pressure levels ($P_x$) for cell loading. The distance cells travel into the channels, and the applied mechanical deformation is dependent upon the pressure differential ($\Delta P$) generated between the channel inlet and outlet.

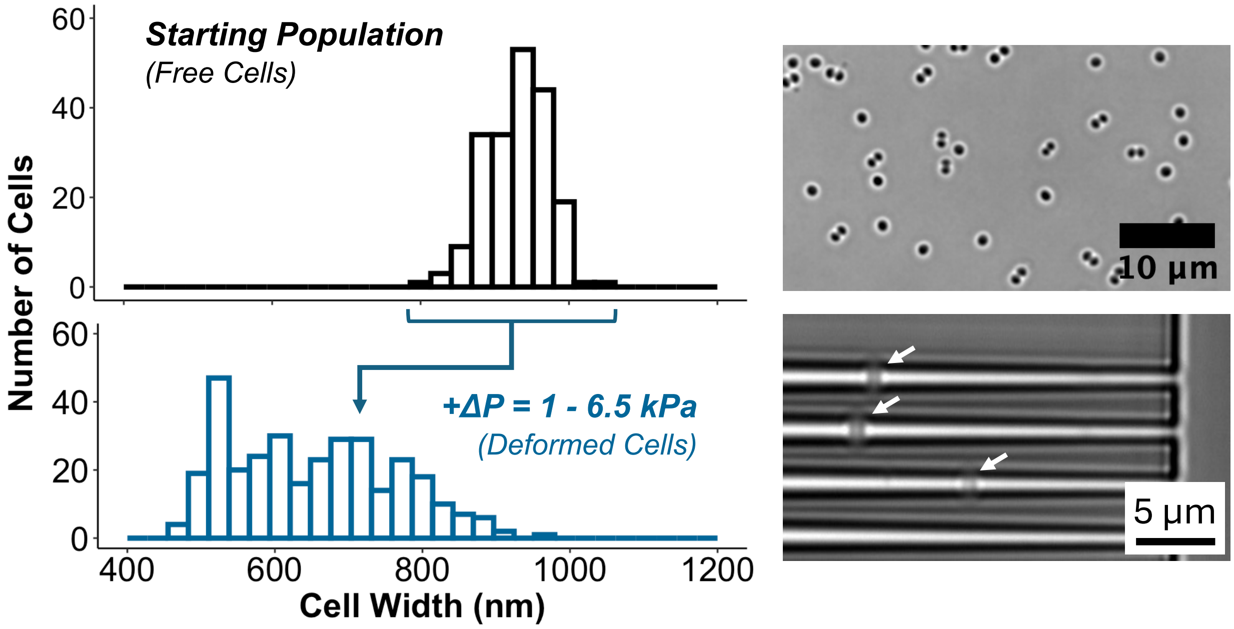


**Figure S2:** Distribution of undeformed (n = 199) and deformed (n = 322) cell width of *S. aureus*. Representative image of *S. aureus* cells on agarose pads (top) and within tapered channels (bottom, white arrows) is also shown.

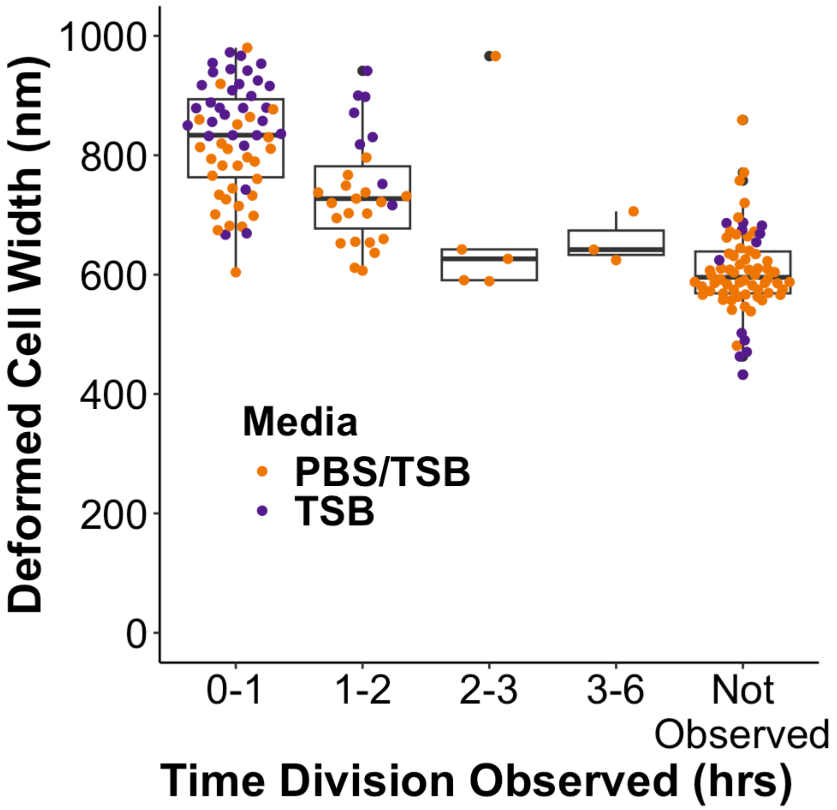


**Figure S3.** Time to division for single, COL EzrA-sGFP cells within the extrusion loading device. Cells were loaded in TSB (purple) or 50:50 TSB:PBS (orange) to reduce background fluorescence for imaging. Media composition appeared to have no influence on the observed relationship between cell division time and deformation, where greater deformation appears to slow or inhibit cell growth. (N =164 cells from 3 replicate experiments).

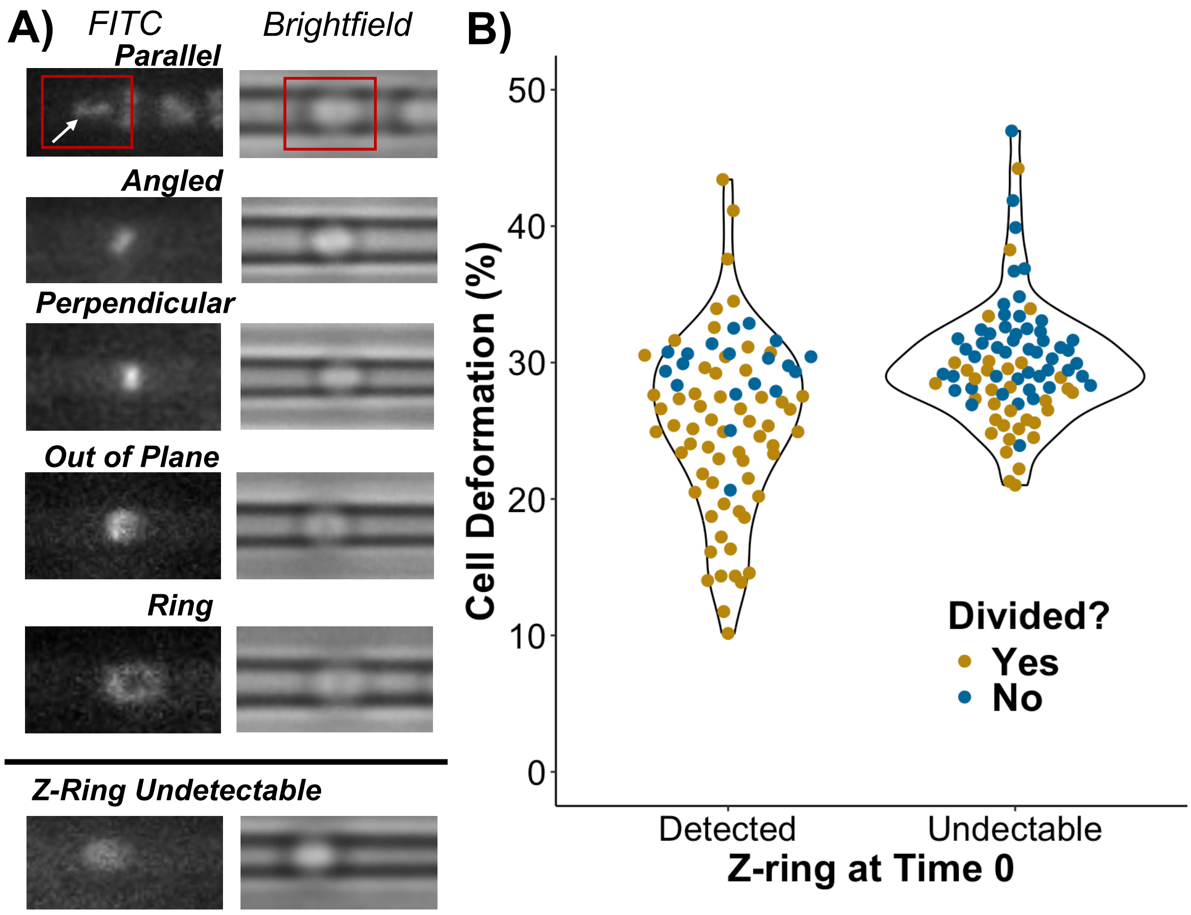


**Figure S4.** Representative fluorescence and brightfield images showing possible Z-ring orientations for cells within the extrusion loading device (A). In some instances, Z-rings were undetectable at time 0, and cells instead exhibited uniform, low-intensity fluorescence across the entire cell. Cells are grouped based on Z-ring orientation at time 0 and further classified into dividing (yellow) and non-dividing (blue) cells (N = 164 from 3 replicate experiments) (B).

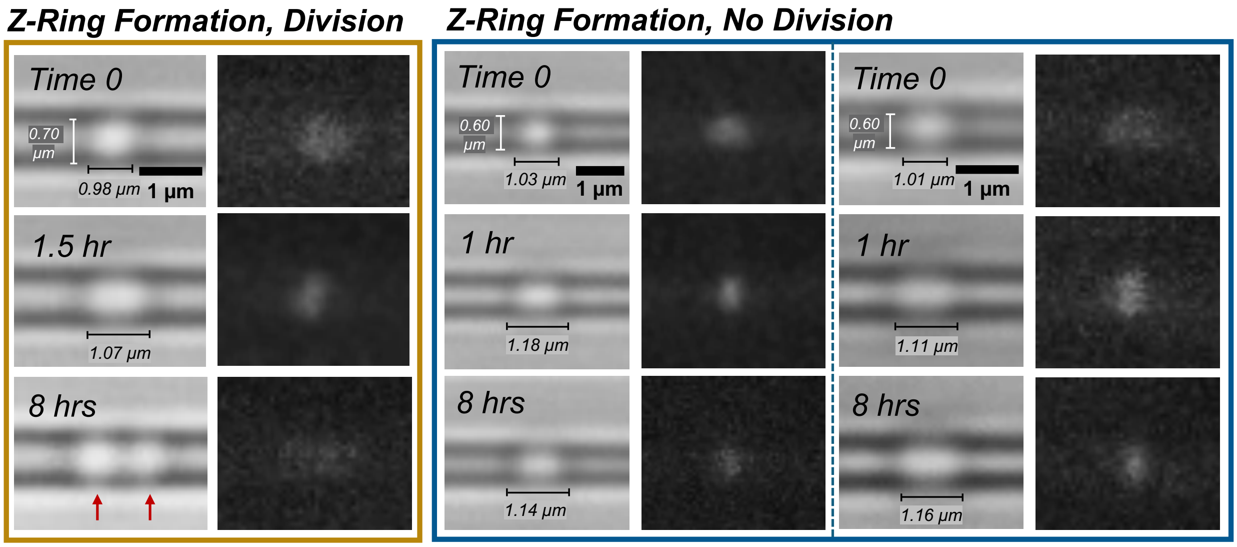


**Figure S5.** Representative images illustrating Z-ring formation and elongation in cells subjected to mechanical deformation within the extrusion loading device. Instances where division both was (left, gold box) and was not (right, blue box) observed over the course of an 8-hour experiment are shown (red arrows mark daughter cells after division).

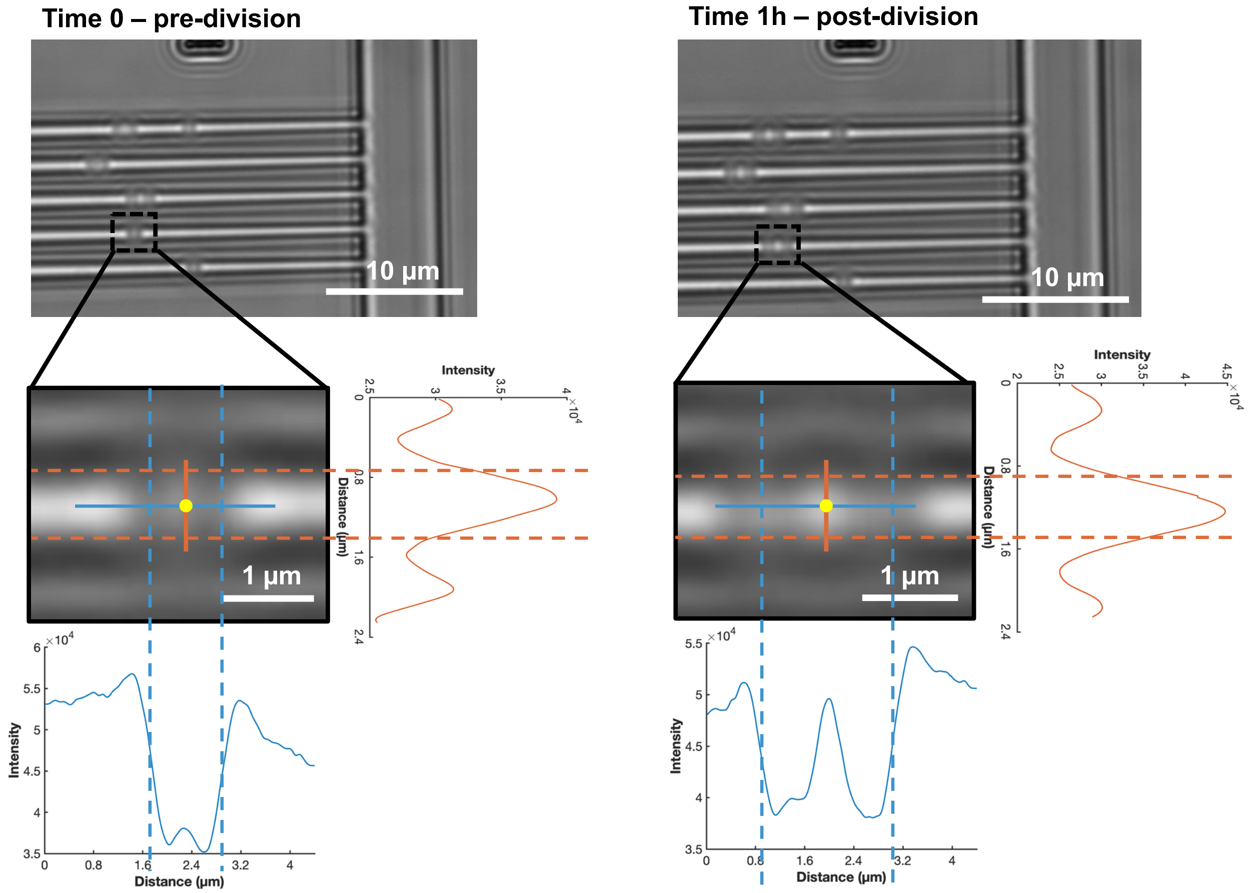


**Figure S6.** Representative images of cells pre- and post-division in tapered channels. Region of interest containing cell(s) is magnified and vertical (blue) and horizontal (orange) intensity traces are shown to identify cell boundaries and midpoint (yellow circle).
